# Genetic association studies using disease liabilities from deep neural networks

**DOI:** 10.1101/2023.01.18.23284383

**Published:** 2023-01-19

**Authors:** Lu Yang, Marie C. Sadler, Russ B. Altman

**Affiliations:** 1 Deparment of Bioengineering, Stanford University, Stanford, CA, 94305, USA; 2 Department of Genetics, Stanford University, Stanford, CA, 94305, USA; 3 Department of Medicine, Stanford University, Stanford, CA, 94305, USA; 4 Department of Computer Science, Stanford University, Stanford, CA, 94305, USA; 5 University Center for Primary Care and Public Health, Lausanne, 1010, Switzerland; 6 Swiss Institute of Bioinformatics, Lausanne, 1015, Switzerland

**Keywords:** GWAS, complex traits, polygenic risk scores, AI-based genetic prediction, disease liability

## Abstract

The case-control study is a widely used method for investigating the genetic landscape of binary traits. However, the health-related outcome or disease status of participants in long-term, prospective cohort studies such as the UK Biobank are subject to change. Here, we develop an approach for the genetic association study leveraging disease liabilities computed from a deep patient phenotyping framework (AI-based liability). Analyzing 44 common traits in 261,807 participants from the UK Biobank, we identified novel loci compared to the conventional case-control (CC) association studies. Our results showed that combining liability scores with CC status was more powerful than the CC-GWAS in detecting independent genetic loci across different diseases. This boost in statistical power was further reflected in increased SNP-based heritability estimates. Moreover, polygenic risk scores calculated from AI-based liabilities better identified newly diagnosed cases in the 2022 release of the UK Biobank that served as controls in the 2019 version (6.2% percentile rank increase on average). These findings demonstrate the utility of deep neural networks that are able to model disease liabilities from high-dimensional phenotypic data in large-scale population cohorts. Our pipeline of genome-wide association studies with disease liabilities can be applied to other biobanks with rich phenotype and genotype data.

## Introduction

The advent of biobanks has greatly reduced the cost and difficulty of collecting large genome-wide data^1,2^, and have enabled a more comprehensive understanding of the genetic basis of human health and disease^3^. The UK Biobank (UKBB) database has genotype data for about 500,000 participants and their socio-demographic, lifestyle and health-related phenotypes^4^. It provides a cohort of unparalleled size and scope, allowing the studies of human genetics research^5,6^. Over the past 15 years, genome-wide association studies (GWAS) of continuous and binary traits have provided new insights into the genetic architecture of a wide range of complex diseases^5^. However, while large-scale genotyping helps identify and localize common-to-rare variant signals, the proportion of phenotypic variance captured is limited^7^. In addition, recent studies have reported that the heritability estimates $\left(h_{SNP}^{2}\right)$ based on pedigree studies are higher than the ones computed from SNP-based GWAS hits^8,9^. This gap also labeled as “missing heritability” is substantial and can be due to multiple factors, such as sample size, omitting rare and structural variants, as well as gene-environment interactions^10^.

To unravel genetics’ role in disease studies, researchers typically compare patients with the disease (the cases) to individuals who are not (the controls). However, patient cohorts may progress beyond a dichotomous setting of “diseased”and “non-diseased”. Healthy patients can become diseased years down the line and GWAS studies explicitly modelling age-at-onset showed an increase in statistical power compared to case-control (CC) studies^11–13^. Time-to-event analyses are computationally expensive and alternative approaches have focused on integrating family history to capture the underlying disease liability. Genome-wide association study by proxy (GWAX) where individuals who reported cases within family members are included as cases themselves can identify new risk loci^14^. GWAX has been particularly popular in the study of Alzheimer’s disease where 46,828 proxy-cases in addition to 2,447 diagnosed cases in the UKBB contributed to increased statistical power^15,16^. Going beyond a binary phenotype, more refined models have used family history to derive a continuous genetic liability phenotype where CC and family history configurations are differentiated more appropriately^17^.

Although previous studies have identified thousands of disease-associated loci, researchers have yet to deeply explore the joint contribution of family history, clinical measures, and lifestyle factors to model disease liabilities. Machine learning techniques have advanced in recent years, allowing to reveal patterns in massive, high-dimensional datasets and capturing non-linear relationships^18,19^. Our study seeks to improve genetic prediction with deep learning-based estimates of disease liability probabilities that leverage joint semantic and structure-based embeddings of phenotypes in the UKBB. To the best of our knowledge, this is the first demonstration of leveraging deep neural networks to present disease phenotypes quantitatively for genetic studies of complex traits.

Recently, we developed a framework POPDx^20^ to recognize a comprehensive set of phenotype labels in UK Biobank. Based on this framework, we present a methodology to improve the genetic prediction of complex traits using a continuous representation of the phenotypes based on individual-level data in the UKBB. Applied to 44 complex traits, our method improved statistical power in GWASs as demonstrated through higher heritability estimates, and higher polygenic risk score (PRS) for newly diagnosed cases. We also found many putative disease susceptibility variants for phenotypes such as essential hypertension, breast neoplasm, uterine leiomyoma, hypothyroidism, type 2 diabetes, alcoholism, myocardial infarction, and brain cancer compared to traditional CC studies.

## Results

### AI-based pipeline for genetic studies of complex diseases

**Figure 1.**
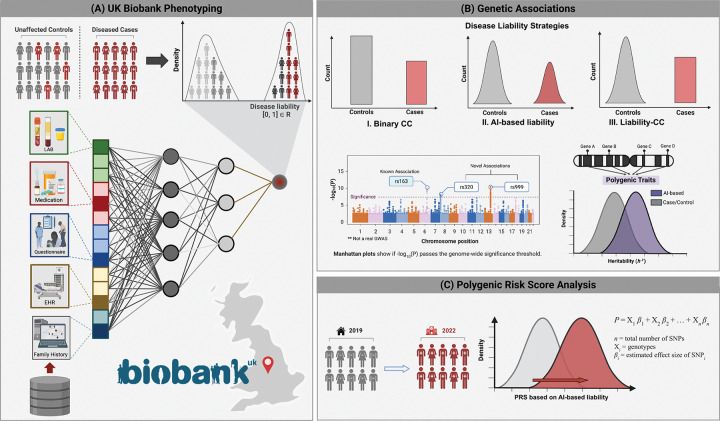
Overview of our AI-based genetic analysis. (A) Patient phenotyping in UK Biobank is the first step in this study’s workflow. After extracting the data, we preprocess diverse patient information such as laboratory results, medication intake, answered questionnaires, medical records, and family history. This probability distribution for each disease of interest is obtained by using an AI-based framework to train the one-hot encoding matrix of patient embeddings with the corresponding binary (case-control) diagnostic labels. The outputs of the patient phenotyping framework are continuous values between 0 and 1, representing the probabilities of complex traits that belong to an individual (disease liability). (B) Three different methods are used to conduct genome-wide association studies of 44 complex traits, including (i) case and control status of the patient (CC), (ii) AI-based disease liabilities, (iii) combined CC and AI-based disease liabilities. SNP-heritability estimates of 44 traits are computed based on the GWAS summary statistics. (C) We identify new cases of diseases based on the 2019 and 2022 releases of phenotypic data and then compare the polygenic risk scores of these newly identified cases calculated from the AI-based liabilities and case-control status of the patients. Figure created with BioRender.com.

We used a deep learning framework^20^ to extract multiple layers of information, including electronic medical records, laboratory tests, physical measurements, answered questionnaires and interviews about the socio-demographic status, lifestyle, and family history of 392,246 individuals in the UK Biobank. The overview of our artificial intelligence (AI)-based genetic analysis is illustrated in Figure 1. First, we used a pre-trained deep learning framework (POPDx)^20^ for patient phenotyping in UK Biobank. The performance of the patient phenotyping is shown in Table 1. The AI-based method transformed the binary Phecode labels of the patients into a continuous spectrum that represents the probability of disease assigned to each individual as shown in Figure 1A. Thus, we retrieved the output of POPDx as the disease liability scores for downstream genetic analysis instead of the conventional CC strategy of disease studies. In the next step, we conducted GWAS analyses for 44 common traits using the AI-based disease liability scores on 577,395 directly genotyped SNP markers. Then, we compared the disease-associated risk loci identified by the AI-based approach to the associations from the ascertained cases and controls routine (Figure 1B). In addition, we estimated the heritability scores $\left(h_{SNP}^{2}\right)$ of these phenotypes using the summary statistics from the AI-based and CC GWAS. Lastly, we estimated PRS effects using the liability scale as well as the original CC status. We then focused on new cases that have emerged in a more recent release of the UKBB and compared their PRS percentiles obtained from the AI-based and CC phenotypes. Overall, we seek to quantify the extent to which AI-based phenotyping helps genetic prediction by finding new disease variants, improving heritability estimates, and enhancing PRS derivations.

### New risk loci identified in GWAS

**Figure 2.**
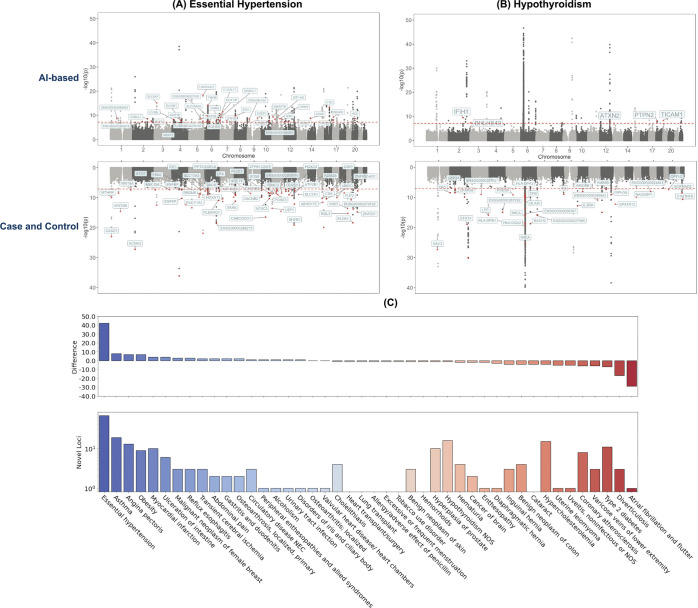
GWAS analysis using the AI-based disease liabilities. Mirrored Manhattan plots of GWAS results for (A) essential hypertension and (B) hypothyroidism. The CC-GWAS results are shown in the bottom panel with annotated risk loci. The GWAS analyses conducted through the AI-based disease liability are shown in the top panel with highlighted risk loci that are not significant in the CC-GWAS. Chromosome position is plotted in genomic order on the x-axis. The y-axis indicates the strength of the association (P values). The dashed horizontal line in red denotes the genome-wide significance threshold of 5×10^−8^. (C) Comparing the number of novel risk loci identified in the AI-based approach with the case-control (CC) associations across 44 complex traits. The top panel illustrates the difference in risk loci counts in descending order (blue to red). The lower panel shows the number of independent variants only significant in the AI-based approach.

**Figure 3.**
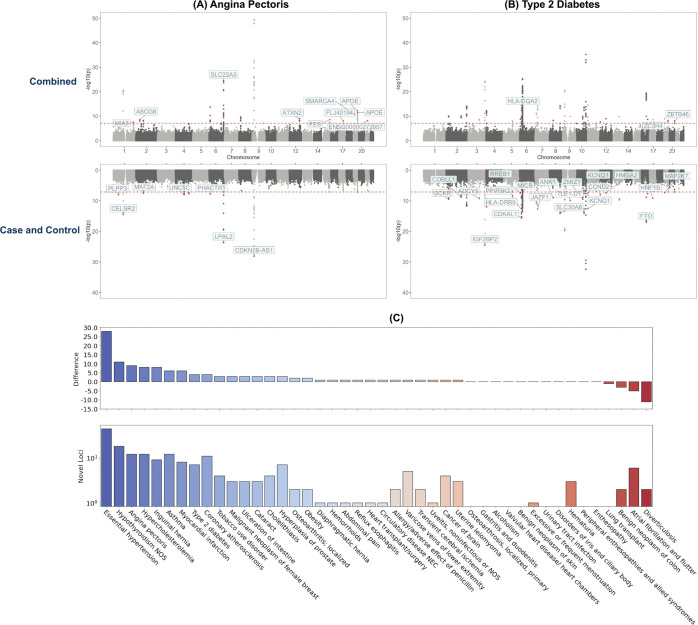
GWAS analysis using the combination of cases and AI-based disease liabilities of the controls. Mirrored Manhattan plots of GWAS results for (A) angina pectoris and (B) type 2 diabetes. The CC-GWAS results are shown in the lower panel with annotated risk loci. The GWAS analyses conducted via the liability-CC method are shown in the upper panel with highlighted risk loci that are not significant in the CC-GWAS. Chromosome position is plotted in genomic order on the x-axis. The y-axis indicates the strength of the association (P values). The dashed horizontal line in red denotes the genome-wide significance threshold of 5×10^−8^. (C) Comparing the number of novel risk loci identified in the combined approach with the case-control (CC) associations across 44 complex traits. The upper panel illustrates the difference in risk loci counts in descending order (blue to red). The lower panel shows the number of independent variants only significant in the combined approach.

We leveraged AI-based disease liabilities to conduct GWAS analyses and discovered independent risk loci for a wide range of complex and polygenic traits. Three methods of patient characterization were used to identify disease-associated risk loci: (1) cases and controls defined by the in-patient diagnosis record of the UK Biobank dataset (CC), (2) AI-based disease liabilities, (3) a combination of AI-based disease liabilities and case-control status (combined CC-liability). Figure 2 compares the AI-based method and the CC GWAS study using Manhattan plots of two common traits with highlighted risk loci. We identified 772 genome-wide significant SNPs in 114 distinct loci for essential hypertension, compared to 299 significant SNPs in 100 unique loci in the CC analysis (Figure 2A). Figure 2B depicts the GWAS analysis for hypothyroidism, one common thyroid disorder. In addition, we identified 1,932 genome-wide significant associations at 42 distinct loci using AI-based liabilities. The novel loci link the interferon-induced helicase gene (IFIH1), a helix-loop-helix protein gene (BHLHE40), ataxin 2 gene (ATXN2), protein tyrosine phosphatase N2 gene (PTPN2), and an adaptor protein-coding gene in the TLR signaling pathways (TICAM2) to hypothyroidism. Generally, our AI-based findings indicate an increased capability to discover additional genome-wide significant SNP associations in many complex traits (Figure 2C). In addition, the AI-based approach revealed new variants for 37 out of 44 phenotypes that the conventional analyses have missed under a similar experimental condition (sample size, etc.). The AI-based method associates fewer risk loci for specific phenotypes compared to CC-GWAS. However, it still assists in identifying additional risk loci, such as CHRDL2 (Chordin Like 2) and CABLES2 (Cdk5 and Abl enzyme substrate 2) associated with diverticulosis, a disease that affects the large intestine. CHRDL2 has previously been shown to be upregulated in colorectal cancer (CRC) tissues^21^. According to earlier research, CABLES2 has a crucial function in colorectal carcinogenesis^22^. Figure 3 illustrates the improvement of multiple-traits GWAS by combining diagnosed cases with AI-based disease liabilities of the controls. In this scenario, controls are assigned disease liability probabilities (range of [0,1]) whereas cases are assigned a probability of 1. Manhattan plots (Figure 3A) are shown to summarize GWAS results for angina pectoris and type 2 diabetes. We annotate a few genetic variants highly associated with these two traits, but some SNP-gene pairs are only found in the combined approach. Through a combination of cases with AI-based disease liabilities of the controls, we identified more genome-wide risk loci for 29 out of 44 phenotypes compared to the conventional GWAS analyses. ABCG8 (ATP-binding cassette subfamily G member 8) is an instance of a new susceptibility gene for angina pectoris identified by our combined approach. It has been previously reported that ABCG8 variants are risk factors for coronary heart disease^23^. ZC3H4 (zinc finger CCCH domain-containing protein 4) is a candidate gene associated with susceptibility to type 2 diabetes (T2D) identified via the combined approach alone. zinc finger protein. Previous research indicates ZC3H4 having an essential role in mediating non-coding transcription in human cells^24^. It was also previously reported the association ZC3H4 and uncontrolled eating^25^. Additional results from the GWAS studies are provided in the Supplementary Figures 1, 2 and Supplementary Table ST1.

We performed an in-depth investigation of our GWAS results (Supplementary Table GWAS Significant Loci). Most of the significant loci identified by the liability and CC-liability methods have been either reported in our CC analysis or other previous studies. Some SNPs are missed in the CC study but could be validated in the literature. For example, rs6544713 (ABCG8) and rs12190287 (TCF21) identified in our combined approach for coronary atherosclerosis were previously found to be associated with the risk for coronary artery disease^26–28^. For breast cancer (malignant neoplasm of female breast), rs13281615 (PCAT1) is highly associated in both AI-based and combined approach. It was not identified in our CC analysis but was reported in other breast cancer studies^29,30^. Some SNPs such as rs10889338 (DOCK7), rs12916 (HMGCR), rs10455872 (LPA), rs15285 (LPL), rs507666 (ABO), and rs11057830 (SCARB1) were identified to be associated with hypercholesterolemia using the CC-liability method. These SNPs were found to be significant in the other GWAS studies of the UKBB^31^.

### Increasing heritability estimates

Next, we calculated SNP-based heritability $\left(h_{SNP}^{2}\right)$ estimates of 44 phenotypes using summary statistics from GWAS analysis. Heritability estimates were based on the BLD-LDAK model which assumes that the expected $h_{SNP}^{2}$ depends on linkage disequilibrium (LD), minor allele frequency (MAF) and 66 functional annotations^32^ (Figure 3, Supplementary Table ST2). Across the 44 traits, the average $h_{SNP}^{2}$ based on 577,395 markers was 0.040 for the AI-based phenotype characterization which was significantly higher than the average of 0.030 for the CC analysis (paired two-sided t-test: p-value = 1.77e-4; Figure 3A). The combination of disease liability and known CC status yielded a $h_{SNP}^{2}$ mean of 0.038 across traits which was not significantly different from using the liability scale alone (paired two-sided t-test: p-value = 0.27). Across the 44 traits, heritability estimates were higher for 32 traits when using disease liability scores compared to CC and 43 traits had higher $h_{SNP}^{2}$ when considering the combined CC-liability scores (Figure 3A). On average, the heritability from the combined phenotype characterization was significantly higher than the CC analysis (paired two-sided t-test: p-value = 4.95e-09; Figure 3A).

**Figure 4.**
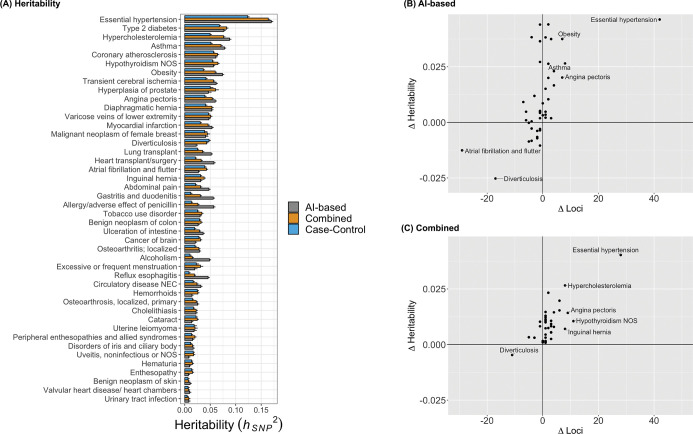
Heritability estimates $\left(h_{SNP}^{2}\right)$ in comparison with identified GWAS loci. (A) Bar plot representing SNP-heritability estimates utilizing three different strategies, including cases-control (blue), combined (orange), and AI-based (grey). (B) We plot the increase in number of independent loci (x-axis) against the increase in heritability estimates by the AI-based approach in comparison with the case-control method. (C) We plot the increase in number of independent loci (x-axis) against the increase in heritability estimates by the combined approach in comparison with the case-control method.

We further compared the difference in $h_{SNP}^{2}$ to the difference in identified independent loci between the liability scale and CC-GWAS. Both quantities highly correlate, with essential hypertension benefiting the most from the liability scale in terms of $h_{SNP}^{2}$ and identified loci (Figure 4B). Comparing the CC-GWAS to the combined CC-liability phenotype definition resulted in similar results (Figure 4C). Using the CC-GWAS as baseline, the number of identified loci, however, was only significantly higher when considering the combined CC-liability phenotype (two-sided Wilcoxon signed rank test across 44 traits: p-value = 4.44e-4) and not with the liability scale alone (two-sided Wilcoxon signed rank test across 44 traits: p-value = 0.35). As an example, 26 loci were identified in the coronary atherosclerosis CC-GWAS, 20 in the liability-scale GWAS, but 30 in the combined CC-liability GWAS.

### Recognizing new cases with AI empowered PRS

**Figure 5.**
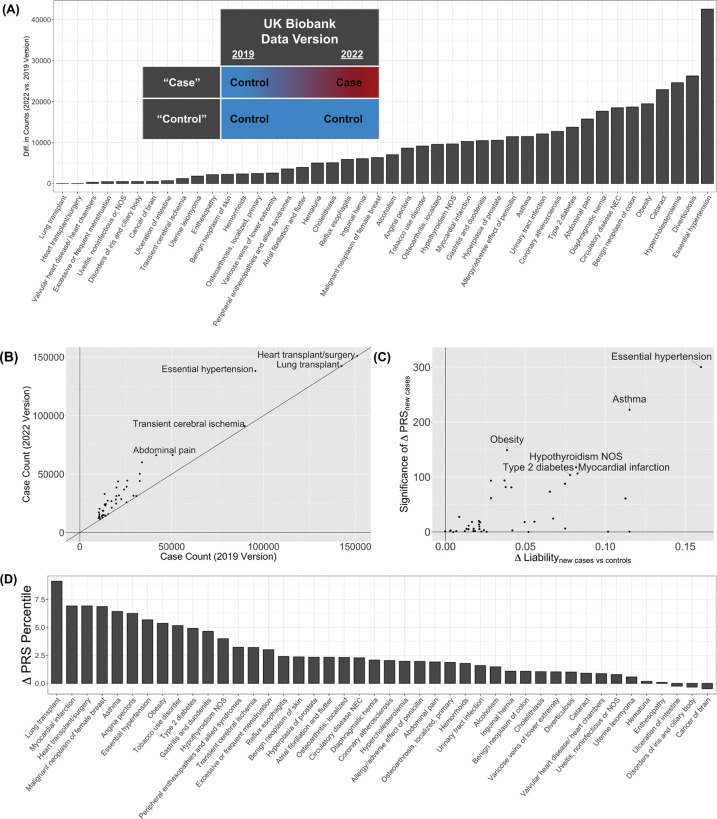
PRS analysis applied on newly diagnosed cases in the UKBB. (A) Bar plot showing the difference in case count per trait between the 2019 and 2022 release of the UKBB. (B) Scatter plot comparing the absolute case count per trait between the two releases. (C) Scatter plot comparing the difference in liability scores between newly diagnosed cases and persistent controls (x-axis) and the significance in PRS difference for new cases between CC and liability estimates (y-axis). The PRS difference results from two-sided Wilcoxon signed-rank tests (see Methods) and is plotted as a positive value if liability PRS allowed to rank newly diagnosed cases higher than CC PRS. (D) Difference in PRS percentile per trait between liability and CC PRS calculations. PRS percentiles were calculated relative to the entire dataset and the difference displayed here was calculated on newly diagnosed cases.

Placing individuals on a continuous spectrum instead of assigning them a binary CC value holds the promise of capturing who is most at risk for developing the disease (Figure 5A). To test this hypothesis, we identified the newly emerged cases in the latest release of UK Biobank diagnosis records (2022) compared to the previous release (2019; Figure 5B). First, we assessed whether liability scores were indeed higher for emerging cases and then tested whether the same holds for polygenic risk scores (PRSs). For the latter, we estimated predictor (i.e. SNP) effects for the CC and liability phenotype definitions separately, and calculated corresponding PRSs. Thus, each individual was assigned two PRSs - one calculated through CC phenotypes and the other through liability scores. Per phenotype, the corresponding PRS ranks of new cases were then compared side-by-side. Liability scores of new cases were higher for 43 out of the 44 traits when averaging among the individuals, and the difference was statistically significant for 37/44 traits at a Bonferroni-corrected threshold of p-value < 0.05/44 (two-sided t-test of liability scores of newly emerged cases vs consistent controls; Supplementary Table ST3). The same trend could be observed on the genetic level (Figure 5C). For 32/44 traits, newly emerged cases significantly ranked higher (i.e. had a higher genetic risk) when the PRS was calculated on the liability scale than with the CC definition (at a p-value < 0.05/44; Method section: Polygenic risk score analysis). When translating the improvement in genetic prediction to PRS percentiles, we found that across diseases new cases had on average a 6.2% percentile rank increase (Figure 5D, Supplementary Table ST4).

## Discussion

We introduced a new framework for genetic association testing where the phenotypes are modelled as disease liabilities derived from deep neural networks. We demonstrated how integrating a variety of patient data—including family history, electronic medical records, socio-demographic characteristics, lifestyle surveys, physical measures, and laboratory examinations—can significantly contribute to boosting genetic association power and prediction performance. The deep neural networks were trained on the entire UKBB cohort, however, computed phenotype probabilities could differ from the true diagnosis status. Nevertheless, raw model outputs resulted in significantly higher genetic heritabilities $\left(h_{SNP}^{2}\right)$ than using binary diagnosis values. For the 44 traits, the number of identified loci was not significantly higher in this setting than in the CC-GWAS, although it helped discover additional loci that the CC-GWAS has missed. The prediction accuracy of phenotype labels was generally very high (mean AUROC of 90%, Table 1). Although AUROC scores did not correlate with the difference in identified loci between liability and CC-GWAS (two-sided Pearson correlation test p-value equalled 0.87 calculated over the 44 traits), performance increased when incorporating the true disease state into the modelled disease liabilities (combined CC-liability). With this approach, both heritability estimates and number of identified GWAS loci were significantly higher compared to the CC status analysis.

Overall, the liability scale performed well for traits such as essential hypertension, hypothyroidism, asthma, angina pectoris, obesity, myocardial infarction and breast cancer. On the other hand, diverticulosis and atrial fibrillation gained little from the disease liability representation of the phenotype data, and performance remained worse compared to CC analyses even with the CC-liability approach. We explored the longitudinal changes associated with patients’ health states by comparing the 2022 release of UK Biobank data sets to an earlier release (Figure 5A, B). Our method provides an effective patient phenotyping algorithm that can differentiate the newly diseased population from the healthy individuals based on the liability scores. Accordingly, the PRS ranks of new cases from the AI-based liability approach are significantly higher than those computed through the prevailing CC method. Taken together, these results indicate the liability phenotypes better capture the underlying genetic architecture than a static dichotomous classification.

Although our AI-based liability and combined CC-liability approaches increased the statistical power of genetic prediction of common traits in the UK Biobank, they have several limitations. First, patient phenotyping is noisy, resulting in incorrect disease liabilities which in turn impairs the downstream genetic analysis. Second, our strategy is specific to the UKBB cohorts; in addition to family history and medical data, our AI-based model uses lifestyle, environmental exposures, and lab examinations to predict patient phenotypes. These data are not available in some observational studies. Third, for computational reasons, we limited the analysis to directly-genotyped SNPs which may explain why the heritability scores for some diseases are lower than those reported in other research studies^33,34^. We have not applied our approaches to other prospective cohort studies, however, the pipeline could be adapted to other biobanks. On a comparable scale, large population cohorts such as the “All of Us” program^35^, China Kadoorie Biobank, and the US Million Veteran Program^36^ can be promising resources to implement our genetic analyses. For all these, the assessment of AI-based disease liabilities may improve the power to detect genetic associations.

## Methods

### UK Biobank data

The UKBB is a cohort with rich phenotype data including International Classification of Diseases, Tenth Revision (ICD-10) codes from which we could derive phecodes^4,37^. Phecodes are curated groups of ICD-10 codes for defining phenotypes^37,38^. We excluded individuals with no available ICD-10 codes in 2019 data version and used remaining individuals (N = 392,246) to infer phecodes and calculate disease liability probabilities (section POPDx). Genetic data analyses were restricted to unrelated white British individuals that passed additional genetic quality control (QC) filters according to the UKBB (*used.in.pca.calculation* = 1 and *in.white.British.ancestry.subset* = 1 in Sample-QC v2 file). Furthermore, samples were excluded if the genetically inferred sex did not match the self-reported sex and if they had withdrawn their consent. Analyses were performed on autosomal genotype data provided by the UK Biobank with MAF > 0.01 (649,644 SNP markers, version 2). Further quality filters and overlapping with external datasets (i.e., SNP annotations) could reduce this number to 577,395 markers. All reported genetic analyses are for 261,807 unrelated white British individuals on *geq* 577,395 genetic markers. Sex-specific traits were analysed on 142,844 females and 118,963 males, respectively.

### Phenotype data

ICD billing codes are commonly utilized to select individuals from large observational datasets such as the UK Biobank. Also, cases of multiple ICD codes are often put together as a group of patients to study a specific disease. Phecodes, combinations of relevant ICD codes used to describe phenotypes, have worked well in clinical research^38^. In this study, we retrieved 44 specific phecodes that associate with more than 10,000 patients to represent 44 common diseases from the 2019 download of phenotype data. To obtain diagnostic labels for all the individuals in the UK Biobank, we mapped International Classification of Diseases Tenth Revision (ICD-10) codes to phecodes^37,39,40^. From the 2019 downloads, we received phenotype data from the UK Biobank. Again, we obtained an updated release of phenotype data in early 2022. On average, participants have more ICD-10 codes in their records. For each of the 44 complex traits, we have identified the individuals that are no longer part of the control group. For PRS validation, the new cases are compared to the controls that do not have the diagnostic code for both data lookups.

### Modelling phenotype probabilities

POPDx is an existing AI-based methodology that outputs the phenotype liability probabilities for each UK Biobank participant^20^. We trained the POPDx framework on the phenotypic and health-related patient data. The 1538 phecodes as gold standard labels correspond to 12,803 ICD-10 diagnosis codes from UKBB. We trained and tested POPDx on all the individuals based on the 2019 version of phenotype data.

### GWAS calculation

We performed GWAS analyses on all 44 complex traits for three phenotype definitions: i) original case-control status, ii) POPDx probability, iii) combined case-POPDx phenotype. GWAS were performed using linear regression as implemented in the LDAK software (v5.2, www.ldak.org^41^). Prior regression, phenotypes were corrected for sex, age, age_2_, and PCs 1–40. The analysis was performed on 649,644 genetic markers with minor allele frequencies (MAF) ≥ 0.01.

We performed clumping on SNPs passing the genome-wide significance threshold of 5e-8 to obtain independent SNP associations (*r*^2^ < 0.05 within a window of 1000kB). Clumping was performed using LDAK and as reference panel we used the genotypes of 20,000 randomly selected individuals among the included 261,807 samples.

### Mapping SNPs to genes

We mapped the SNPs to genes under the guidance of a comprehensive reference annotation (GENCODE GRCh37 release 42)^42^. The GENCODE consortium provides public access to the most recent annotation of human reference genomes^42^. For each SNP, we searched through the genomic locations (genomic start and end positions) of all the protein-coding genes and non-coding RNA genes. The SNPs need to lie within the target genes to be annotated.

### SNP-based heritability estimation

We calculated SNP heritability $\left(h_{SNP}^{2}\right)$ of each trait for each of the three phenotype definitions (see GWAS calculation) using the BLD-LDAK model^32^. BLD-LDAK estimates $h_{SNP}^{2}$ assuming that the expected $h_{SNP}^{2}$ varies with linkage disequilibrium (LD) and minor allele frequency (MAF) while also taking into account 66 functional annotations. Estimates were calculated using SumHer (part of the LDAK software) and pre-computed tagging files designed for use with LDAK v5.2. Provided tagging files were computed on 2,000 white British individuals from the UK Biobank and we chose the set of non-ambiguous directly genotyped SNPs (MAF > 0.01, 577,525 markers of which 577,395 overlapped with our GWAS summmary statistics).

### Polygenic risk score analysis

For each of the 261,807 individuals included in our analyses we calculated polygenic risk scores (PRS) for each trait. Per trait, two PRS were assigned to each person - the first based on original case-control status and the second based on POPDx probabilities. Since our aim was to compare which of the two phenotype definitions assigns a lower percentile rank (i.e., higher PRS) to emerging disease cases, estimation of per-predictor effect sizes and phenotype prediction were performed on the same individuals. Information as to whether an individual turns into a case is not available to either phenotype definition, and thus comparison at a later time point constitutes an independent validation analysis.

Estimation of per-predictor effect sizes was done on individual-level data (N = 261,807) following the LDAK-Bolt-Predict procedure as implemented in the LDAK software v5.2^33^. First, we estimated per-predictor heritabilities which requires the summary statistic of the trait and phenotype definition of interest (see GWAS calculation), a SNP tagging file and corresponding heritability matrix. The SNP tagging file is calculated according to the assumed underlying heritability model which is the BLD-LDAK model in the LDAK-Bolt-Predict procedure. This model makes use of 65 functional annotations of which 64 can be downloaded. The last annotation, also called LDAK weightings, takes into account the LD structure and was calculated on the genetic data of a subset of 5,000 randomly selected individuals (as recommended to reduce computational demands). The tagging file and heritability matrix were then calculated with recommended settings (power = 0.25, window-kb = 1200) on the same subset of 5,000 individuals, and per-predictor heritabilities were derived by integrating the summary statistics data. Next, per-predictor effect sizes were estimated with the bolt predict command in a cross-validation (cv) fashion (suggested cv-proportion of 0.1). Effect size estimation was done using individual-level genetic data of the entire dataset (N = 261,807), covariate-adjusted phenotypes (see GWAS calculation) and the per-predictor heritabilities. The tagging file and heritability matrix were the same in each calculation, but per-predictor heritabilities and estimated effect sizes were specific to each trait and respective phenotype definition (i.e., POPDx probability or original case-control status). Finally, we estimated PRS for each of the 261,807 individuals by projecting their genetic data onto the estimated effect sizes.

Per trait, we compared the two phenotype definitions by concentrating on the newly emerged cases (control in the earlier release and case in the new release). To this end, we first ranked the individuals relative to their PRS in the entire dataset. Each new case would thus have two ranks, once calculated from POPDx probabilities and once from the original case-control classification. For each trait, the difference in ranks of the new cases were then compared through a two-sided Wilcoxon signed-rank test.

## Supplementary Material

Supplement 1

Supplement 2

## Figures and Tables

**Table 1.** AUROCs of Patient phenotyping using the POPDx framework

Phenotypes	Phecode	AUROC

Malignant neoplasm of female breast	174.11	0.9929
Varicose veins of lower extremity	454.1	0.9739
Valvular heart disease/ heart chambers	747.12	0.9661
Myocardial infarction	411.2	0.9605
Hematuria	593.0	0.9562
Excessive or frequent menstruation	626.12	0.9535
Coronary atherosclerosis	411.4	0.9525
Hypothyroidism NOS	244.4	0.9489
Uterine leiomyoma	218.1	0.9464
Hyperplasia of prostate	600.0	0.9440
Angina pectoris	411.3	0.9440
Peripheral enthesopathies and allied syndromes	726.0	0.9431
Cholelithiasis	574.1	0.9425
Asthma	495.0	0.9386
Ulceration of intestine	556.1	0.9340
Type 2 diabetes	250.2	0.9325
Benign neoplasm of colon	208.0	0.9281
Essential hypertension	401.1	0.9258
Cancer of brain	191.11	0.9196
Inguinal hernia	550.1	0.9167
Cataract	366.0	0.9059
Atrial fibrillation and flutter	427.2	0.9056
Osteoarthrosis, localized, primary	740.11	0.8984
Hypercholesterolemia	272.11	0.8970
Circulatory disease NEC	459.9	0.8926
Disorders of iris and ciliary body	379.5	0.8823
Uveitis, noninfectious or NOS	371.1	0.8798
Diverticulosis	562.1	0.8794
Benign neoplasm of skin	216.0	0.8700
Enthesopathy	726.1	0.8698
Obesity	278.1	0.8664
Transient cerebral ischemia	433.31	0.8645
Urinary tract infection	591.0	0.8637
Osteoarthritis; localized	740.1	0.8547
Tobacco use disorder	318.0	0.8539
Hemorrhoids	455.0	0.8511
Lung transplant	510.2	0.8376
Reflux esophagitis	530.14	0.8341
Gastritis and duodenitis	535.0	0.8333
Heart transplant/surgery	429.1	0.8317
Diaphragmatic hernia	550.2	0.8300
Abdominal pain	785.0	0.7981
Alcoholism	317.1	0.7793
Allergy/adverse effect of penicillin	960.2	0.7488
