# Supplementary material for: Genetic association studies using disease liabilities from deep neural networks
Source: medRxiv. 2023 Jan 19:2023.01.18.23284383. Preprint. [Version 1] doi: 10.1101/2023.01.18.23284383 (PMC9882423; doi:10.1101/2023.01.18.23284383)

medRxiv preprint doi: https://doi.org/10.1101/2023.01.18.23284383; this version posted January 19, 2023. The copyright holder for this preprint (which was not certified by peer review) is the author/funder, who has granted medRxiv a license to display the preprint in perpetuity.
It is made available under a CC-BY 4.0 International license .

Supplementary Figure 1. Mirrored Manhattan plots of GWAS analysis using the AI-based disease liabilities. Mirrored Manhattan plots of GWAS results for (A) cancer of Brain, (B) obesity, (C) disorders of iris and ciliary body, (D) myocardial infarction, (E) transient cerebral ischemia, and (F) ulceration of intestine. The CC-GWAS results are shown in the bottom panel with annotated risk loci. The GWAS analyses conducted through the AI-based disease liability are shown in the top panel with highlighted risk loci that are not significant in the CC-GWAS. Chromosome position is plotted in genomic order on the x-axis. The y-axis indicates the strength of the association (P values). The dashed horizontal line in red denotes the genome-wide significance threshold of 5×10^{-8}.

Supplementary Figure 2. Mirrored Manhattan plots of GWAS analysis using the combination of cases and AI-based disease liabilities of the controls. Mirrored Manhattan plots of GWAS results for (A) uterine Leiomyoma, (B) hypercholesterolemia, (C) uveitis, (D) coronary atherosclerosis, (E) varicose veins of lower extremity, and (F) inguinal hernia. The CC-GWAS results are shown in the lower panel with annotated risk loci. The GWAS analyses conducted via the liability-CC method are shown in the upper panel with highlighted risk loci that are not significant in the CC-GWAS. Chromosome position is plotted in genomic order on the x-axis. The y-axis indicates the strength of the association (P values). The dashed horizontal line in red denotes the genome-wide significance threshold of 5×10^{-8}.

medRxiv preprint doi: https://doi.org/10.1101/2023.01.18.23284383; this version posted January 19, 2023. The copyright holder for this preprint
(which was not certified by peer review) is the author/funder, who has granted medRxiv a license to display the preprint in perpetuity.
It is made available under a CC-BY 4.0 International license .

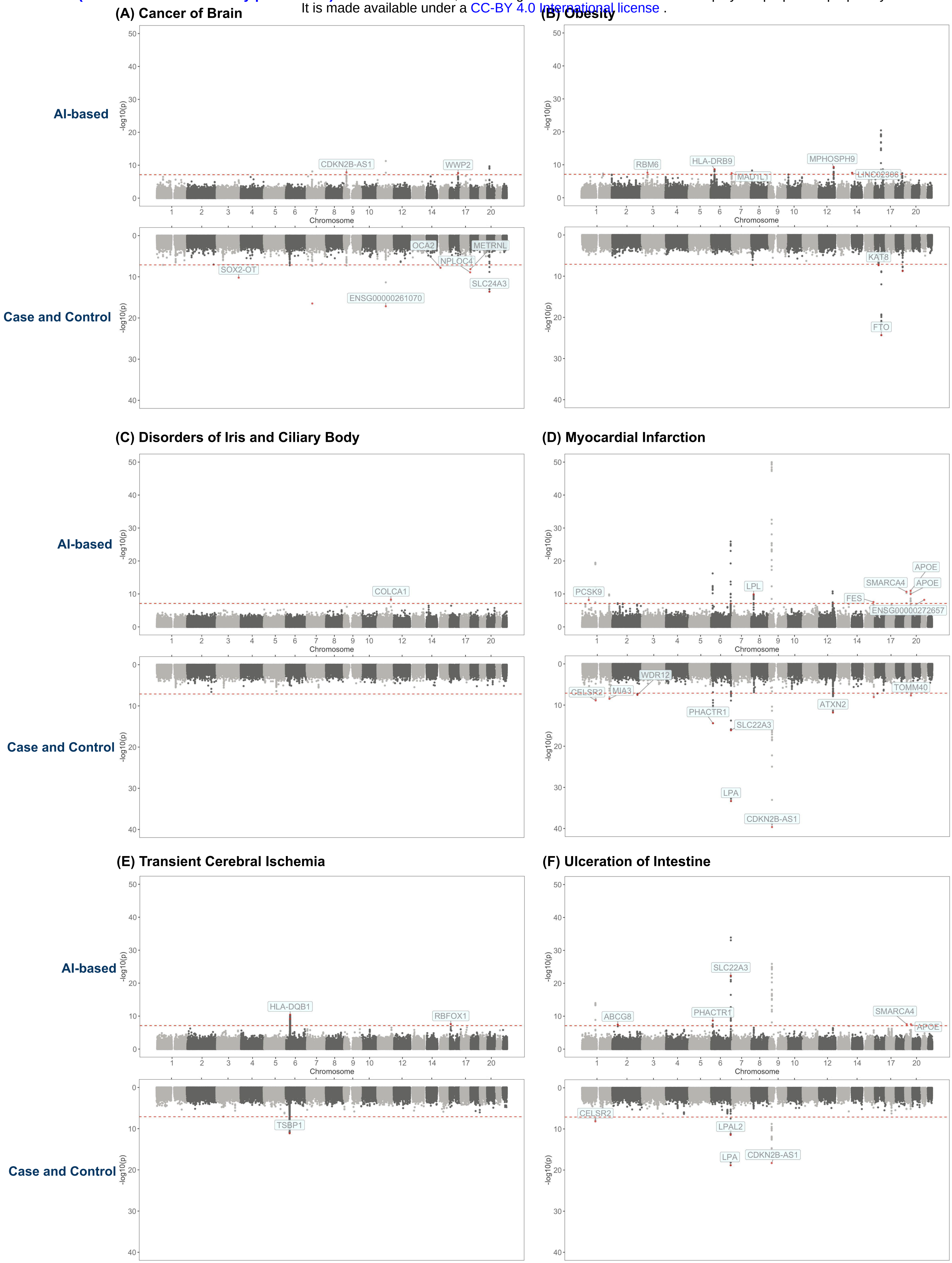

medRxiv preprint doi: https://doi.org/10.1101/2023.01.18.23284383; this version posted January 19, 2023. The copyright holder for this preprint (which was not certified by peer review) is the author/funder, who has granted medRxiv a license to display the preprint in perpetuity.
It is made available under a CC-BY 4.0 International license .

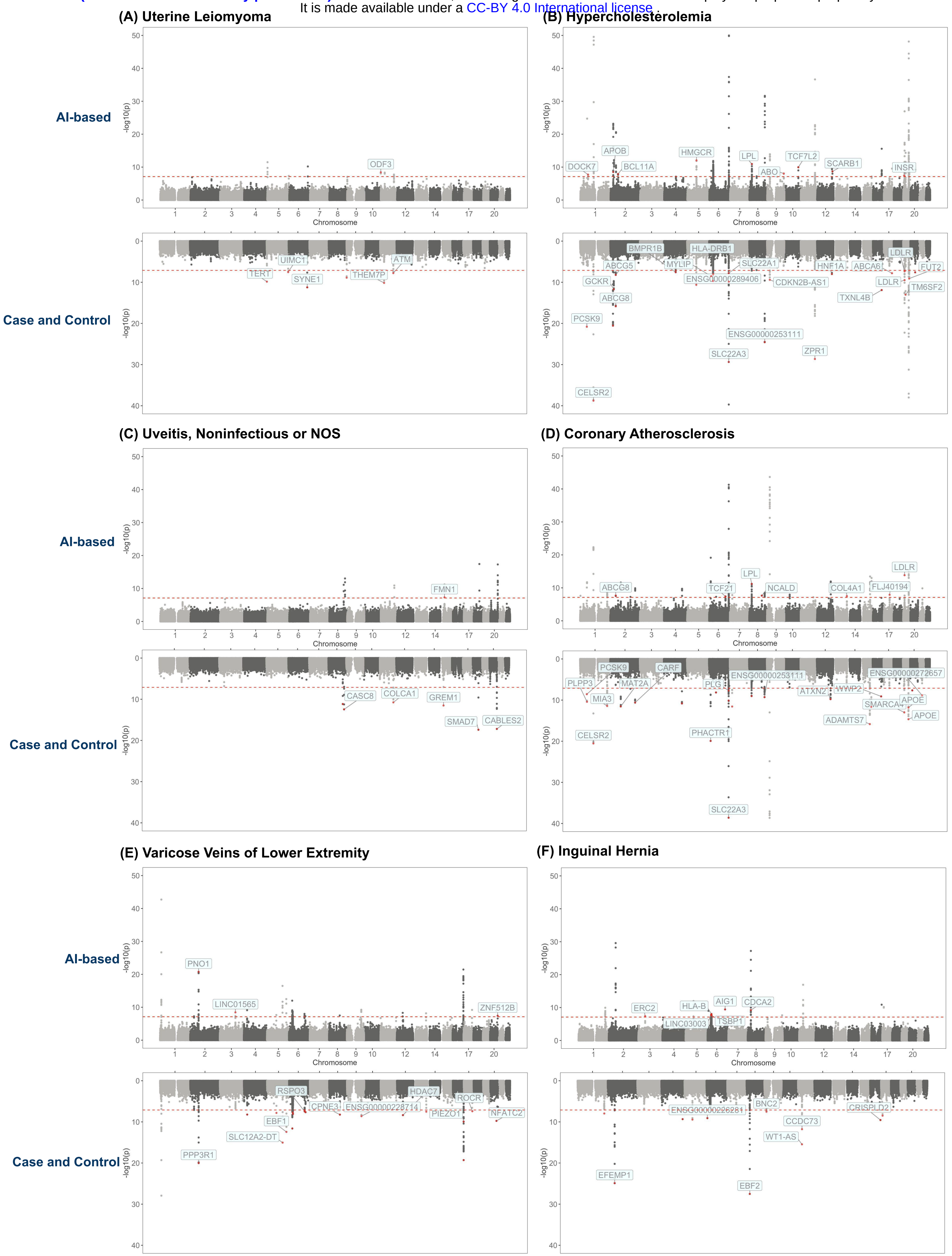

Supplement: Supplement 2 [file NIHPP2023.01.18.23284383v1-supplement-2.pdf]
